# Spinal cord grey matter segmentation challenge

**DOI:** 10.1016/j.neuroimage.2017.03.010

**Published:** 2017-05-15

**Authors:** Ferran Prados, John Ashburner, Claudia Blaiotta, Tom Brosch, Julio Carballido-Gamio, Manuel Jorge Cardoso, Benjamin N. Conrad, Esha Datta, Gergely Dávid, Benjamin De Leener, Sara M. Dupont, Patrick Freund, Claudia A.M. Gandini Wheeler-Kingshott, Francesco Grussu, Roland Henry, Bennett A. Landman, Emil Ljungberg, Bailey Lyttle, Sebastien Ourselin, Nico Papinutto, Salvatore Saporito, Regina Schlaeger, Seth A. Smith, Paul Summers, Roger Tam, Marios C. Yiannakas, Alyssa Zhu, Julien Cohen-Adad

**Affiliations:** a Translational Imaging Group, Centre for Medical Image Computing (CMIC), Department of Medical Physics and Bioengineering, University College London, Malet Place Engineering Building, London WC1E 6BT, UK; b NMR Research Unit, Queen Square MS Centre, Department of Neuroinflammation, UCL Institute of Neurology, University College London, Russell Square, London WC1B 5EH, UK; c Brain MRI 3T Centre, C. Mondino National Neurological Institute, Pavia, Italy; d Dementia Research Centre, Department of Neurodegenerative Disease, UCL Institute of Neurology, University College London, Queen Square, London WC1N 3BG, UK; e NeuroPoly Lab, Polytechnique Montreal, Montreal, QC, Canada; f Functional Neuroimaging Unit, CRIUGM, Université de Montréal, Montreal, QC, Canada; g Department of Medicine, University of British Columbia, Vancouver, BC, Canada V6T 2B5; h Department of Electrical and Computer Engineering, University of British Columbia, Vancouver, BC, Canada V6T 1Z4; i Department of Radiology, UBC MS/MRI Research Group, University of British Columbia, Vancouver, BC, Canada V6T 2B5; j Department of Radiology, European Institute of Oncology, University of Modena and Reggio Emilia, 41121, Modena, MO, Italy; k Department of Radiology and Radiological Sciences, Biomedical Engineering, Ophthalmology, Institute of Imaging Science, Vanderbilt University, Nashville, TN, USA; l Institute of Imaging Science, Vanderbilt University, Nashville, TN, USA; m Wellcome Trust Centre for Neuroimaging, University College London, Queen Square, London WC1N 3BG, UK; n Spinal Cord Injury Center Balgrist, University Hospital Zurich, University of Zurich, Switzerland; o Eindhoven University of Technology, Netherlands; p Department of Neurology, University of California San Francisco, San Francisco, CA, USA; q Department of Electrical Engineering, Computer Science, Biomedical Engineering, Radiology and Radiological Sciences, Institute of Image Science at Vanderbilt University, Nashville, TN, USA; r Department of Brain and Behavioural Sciences, University of Pavia, Italy

**Keywords:** Spinal cord, Grey matter, Segmentation, MRI, Challenge, Evaluation metrics

## Abstract

An important image processing step in spinal cord magnetic resonance imaging is the ability to reliably and accurately segment grey and white matter for tissue specific analysis. There are several semi- or fully-automated segmentation methods for cervical cord cross-sectional area measurement with an excellent performance close or equal to the manual segmentation. However, grey matter segmentation is still challenging due to small cross-sectional size and shape, and active research is being conducted by several groups around the world in this field. Therefore a grey matter spinal cord segmentation challenge was organised to test different capabilities of various methods using the same multi-centre and multi-vendor dataset acquired with distinct 3D gradient-echo sequences. This challenge aimed to characterize the state-of-the-art in the field as well as identifying new opportunities for future improvements. Six different spinal cord grey matter segmentation methods developed independently by various research groups across the world and their performance were compared to manual segmentation outcomes, the present gold-standard. All algorithms provided good overall results for detecting the grey matter butterfly, albeit with variable performance in certain quality-of-segmentation metrics. The data have been made publicly available and the challenge web site remains open to new submissions. No modifications were introduced to any of the presented methods as a result of this challenge for the purposes of this publication.

## Highlights

• First grey matter spinal cord segmentation challenge.
• Six institutions participated in the challenge and compared their methods.
• Public available dataset from multiple vendors and sites.
• The challenge web site remains open to new submissions.

## Introduction

A large spectrum of (non)-traumatic neurological disorders have been linked with spinal cord grey matter (GM) and white matter (WM) tissue changes (Amukotuwa and Cook, 2015). The spinal cord is a challenging area for magnetic resonance imaging (MRI) (Wheeler-Kingshott et al., 2014, Stroman et al., 2014) due to the small cross-sectional area dimension of the spinal cord, the presence of motion, susceptibility artifacts and, in particular, the complex shape and small area fraction of GM tissue. Recently, Yiannakas et al. (2012) demonstrated the feasibility to distinguish between the WM and GM by performing manual segmentation of the cervical cord using a T1-weighted fast field echo (FFE) data acquired in a 3 T scanner with reasonable acquisition times and an in-plane resolution of 0.5×0.5 mm^2^. More recently, Schlaeger et al., 2014, Schlaeger et al., 2015 also demonstrated that spinal cord GM area was the strongest correlate of disability in multiple sclerosis using multivariate models that included brain GM and WM volumes, fluid-attenuated inversion recovery lesion load, T1 lesion load, spinal cord cross-sectional area (CSA), T2 lesion load, age, sex, and disease duration.

Several semi- or fully-automated segmentation methods have been proposed in the last decade for cervical CSA estimation (Losseff et al., 1996, Hickman et al., 2004, Tench et al., 2005, Zivadinov et al., 2008, Horsfield et al., 2010, McIntosh et al., 2011, Bergo et al., 2012, Chen et al., 2013, de Leener et al., 2014, Asman and Bryan, 2014, Taso et al., 2015, El Mendili et al., 2015, de Leener et al., 2016). While most methods present good performance, interpretation and comparison of results between different methods is seldom possible due to the use of different imaging datasets (usually in-house data), different MRI sequences, different ways to obtain gold standard segmentations (number of raters and consensus mask) and the use of various performance scores (2D/slice-wise or 3D/volumetric). Recent cervical cord CSA segmentation methods have reached a performance close to manual segmentation (de Leener et al., 2014; Asman and Bryan, 2014, El Mendili et al., 2015, de Leener et al., 2016), but accurate GM segmentation remains a challenge. Moreover, there is a lack of publicly available datasets with GM/WM contrast and corresponding ground truth that facilitate a fair and reliable comparison across methods.

A GM spinal cord segmentation challenge was organised in conjunction by four internationally recognised spinal cord imaging research groups (University College London, Polytechnique Montreal, University of Zurich and Vanderbilt University) to test the different performances of various methods, with the aim of characterizing the state-of-the-art in the field according to a pre-defined set of assessment criteria as well as identifying opportunities for future improvement. Several GM spinal cord segmentation methods developed independently by various research groups across the world were compared. These methods were used to segment the same multi-centre and multi-vendor dataset acquired with distinct 3D gradient-echo sequences, which are available to the community at http://cmictig.cs.ucl.ac.uk/niftyweb/challenge, and the obtained results were compared to the manual segmentation performed by 4 raters.

## Material

Participating teams applied their automatic or semi-automatic segmentation algorithms to anatomical MR images of 40 healthy spinal cords. Challenge data was composed by 80 datasets, split in 40 training and 40 test datasets, 20 each acquired at 4 different sites (University College London, Polytechnique Montreal, University of Zurich and Vanderbilt University). See Table 1 for demographic data. Algorithms were evaluated against manual segmentations from four trained raters (one from each site who each analysed all data from all sites) in terms of segmentation accuracy and precision using several validation metrics.

**Table 1:** Demographic data per site, first row: number of healthy controls per site, second row: gender - female (F):male (M); third row: mean age in years. Std: standard deviation.

	**Site 1 – UCL**	**Site 2 – Montreal**	**Site 3 – Zurich**	**Site 4 – Vanderbilt**
*Subjects*	20	20	20	20
*Gender*	14F:6M	11F:9M	6F:14M	7F:13M
*Mean Age (Std)*	44.3 (10.4)	33.7 (17.4)	40.6 (10.4)	28.3 (8.2)

### Data

A multi-centre, multi-vendor dataset of spinal cord anatomical images of healthy subjects was provided. Each site provided images from 20 healthy subjects along with WM/GM manual segmentation masks. The acquisition parameters for each site were the following:

• Site 1, University College London. Acquisition was performed using a 3 T Philips Achieva MRI system with dual-transmit technology enabled for all scans (Philips Healthcare, Best, Netherlands) and the manufacturer's product 16-channel neurovascular coil. All participants were immobilised using a MRI-compatible cervical collar (TalarMade Ltd, Chesterfield, UK). The cervical cord was imaged in the axial-oblique plane (i.e. slices perpendicular to the longitudinal axis of the cord) with the center of the imaging volume positioned at the level of C2-3 intervertebral disc. The MRI acquisition parameters were: fat-suppressed 3D slab-selective fast field echo (3D-FFE) with time of repetition (TR)=23 ms; time of echo (TE)=5 ms, flip angle *α*=7°, field-of-view (FOV)=240×180 mm^2^, voxel size=0.5×0.5×5 mm^3^, NEX=8, 10 axial contiguous slices, scanning time 13:34 min. A 15 mm section of the high-resolution 3D-FFE volumetric scan (i.e. 3 slices) was extracted, with the middle slice passing through the C2/C3 intervertebral disc.
• Site 2, Polytechnique Montreal. Acquisition was performed using a 3 T Siemens TIM Trio, with the body coil used for RF transmission and the 12 channels head coil+4 channels neck coil for RF reception. All participants were immobilised with padding. Axial 2D spoiled gradient echo, TR=539 ms, TE=5.41, 12.56 and 19.16 ms (averaged off-line to create a single image with increased SNR), flip angle *α*=35°, readout bandwidth (BW)=200 Hz per pixel, voxel size=0.5×0.5×5 mm^3^, 10 slices, matrix size of 320×320, R=2 acceleration along RL direction with GRAPPA reconstruction, phase stabilization. Scanning time 4:38 min.
• Site 3, University of Zurich. Scanning was performed on a 3 T Siemens Skyra MRI scanner (Siemens Healthcare, Erlangen, Germany) using a 16-channel radio-frequency receive head and neck coil and radio-frequency body transmit coil. All participants wore an MRI-compatible neck collar (Laerdal Medicals, Stavanger, Norway). A 3D high-resolution optimized T2*-weighted multi-echo sequence (multiple echo data image combination; MEDIC) was applied to acquire five high-resolution 3D volumes of the cervical cord at C2/C3 level. Each volume consisted of twenty contiguous slices acquired in the axial-oblique plane and was obtained with a resolution of 0.5×0.5×2.5 mm^3^ within 2:08 min for each of the five volumes. Following parameters were applied: TE=19 ms, TR=44 ms, FOV=192×162 mm^2^, matrix size=384×324, flip angle *α*=11°, and readout bandwidth=260 Hz per pixel. After data acquisition, zero-interpolation filling was used to double in-plane resolution (0.25×0.25 mm^2^) and the five 3D volumes were averaged in the spatial domain to create a single image with increased SNR.
• Site 4, Vanderbilt University. Imaging was performed on a 3 T whole body Philips scanner (Philips Achieva, Best, Netherlands). A two-channel body coil was used in multi-transmit mode for excitation and a 16-channel SENSE neurovascular coil was used for reception. All participants were immobilised using foam pads around the head between the coil and a foam neck pillow. The sequence consisted of a multi-slice, multi-echo fast field echo (mFFE) acquired in the axial plane with the following parameters: TR=700 ms, TE/*δ*TE=7.2/8.9 ms, FOV=160×160 mm^2^, flip angle *α*=28°, voxel size=0.65×0.65×5 mm^3^ interpolated to 0.29×0.29×5 mm^3^, number of echoes=3, NSA=2, 14 axial contiguous slices, SENSE: RL=2. The resulting scan time was 5:46 min. The centre of the imaging volume was positioned at C3/C4 intervertebral disc.

At all sites, the imaging volume was carefully positioned to ensure comparable results across all scans. Written informed consent was obtained from all participants and the work was approved by the respective institution's local research committee. Table 2 summarises the acquisition parameters of the 4 sequences used in this study.

**Table 2:** A summary of acquisition parameters from each site.

	**Site 1 – UCL**	**Site 2 – Montreal**	**Site 3 – Zurich**	**Site 4 – Vanderbilt**
*Scanner*	3 T Philips Achieva	3 T Siemens TIM Trio	3 T Siemens Skyra	3 T Philips Achieva
*Sequence*	3D Gradient echo	2D spoiled gradient multi-echo	3D multi-echo gradient-echo	3D multi-echo gradient-echo
*TE* (ms)	5	5.41,12.56,19.16	19	7.2,16.1,25
*TR* (ms)	23	539	44	700
*Flip Angle* (deg)	7	35	11	28
*FOV* (mm)	240×180	320×320	162×192	160×160
*Resolution* (mm)	0.5×0.5×5	0.5×0.5×5	0.25×0.25×2.5	0.3×0.3×5
*NEX*	8	1	5	2
*Slices*	10 (3 extracted)	10	20	14
*Time* (m:s)	13:34	4:38	10:40	5:46
*Coil (channels)*	16	12+4	16	16
*Coil type*	Neurovascular	Head+Neck	Neurovascular	Neurovascular
*Acceleration*	–	GRAPPA factor 2	–	SENSE RL=2

### Image quality assessment

Image quality assessments were performed for each site at subject level. Signal-to-noise ratio within WM and contrast-to-noise ratio between GM and WM (Grussu et al., 2015, Yiannakas et al., 2016) were computed as: ${\mathrm{SNR}}_{\mathrm{WM}}=\mu_{\mathrm{WM}}/\sigma_{\mathrm{WM}}$ and $\mathrm{CNR}=|\mu_{\mathrm{WM}}-\mu_{\mathrm{GM}}|/\sqrt{\sigma_{\mathrm{WM}}^{2}+\sigma_{\mathrm{GM}}^{2}}$.

### GM mask delineation

Four expert raters, one per site, working independently, manually segmented the GM and WM masks using different software packages following (Yiannakas et al., 2012) guidelines.

Rater 1 (MY) and rater 3 (GD), first outlined GM manually and subsequently outlined the cord CSA in all subjects using the semi-automated *cord finder* option available with JIM (v. 6.0, Xinapse Systems, Northants, UK; http://www.xinapse.com/). GM and cord CSA JIM masks were converted to NIFTI using the JIM masker tool. Pixels that were at least 50% within ROIs were defined to be inside the mask.

Rater 2 (SMD) and 4 (BL) manually outlined both GM and WM masks. Rater 2 used FSLView (Jenkinson et al., 2012) and Rater 4 used MIPAV http://mipav.cit.nih.gov/.

Additionally, in order to assess rater performance, a consensus segmentation of the four raters was calculated using majority voting. In this paper, consensus is defined as voxels receiving three or more rater votes.

### Evaluation framework

An online automatic evaluation tool was made publicly available as part of NiftyWeb (Prados et al., 2016a) at http://cmictig.cs.ucl.ac.uk/niftyweb/. From this website, training and testing data were publicly available for download. The training dataset contained a total of 40 volumes (18F:22M, mean age 36.33±13.98 years), 10 per site, with the WM and GM spinal cord segmentation from 4 expert raters and a text file with the vertebral level of each slice. The testing dataset contained a total of 40 volumes (20F:20M, mean age 37.10±13.01 years), 10 per site, and a text file with the vertebral level. Participants were required to accept a data usage license agreement prior to downloading the data.

Teams submitted their binary tissue segmentation masks and obtained the performance results automatically for both training or testing datasets. The submitted segmentations were assessed using the validation metrics described in the following section.

The evaluation website will remain open to new submissions. Gold standard segmentations of the testing dataset will remain hidden.

### Validation metrics

A number of quantitative scores were used to validate the quality of the submitted binary segmentations. All evaluations were performed in 3D and, in order to cover the same area/volume, only the slices that were processed by all the raters were taken into account. Manual binary segmentation masks were considered as the ground truth (GT). For each provided mask (PM) by the teams, each voxel was classified as: True positive (TP), if it was a GM voxel in GT mask and it was segmented as GM; true negative (TN), if it was a non-GM voxel in GT mask and it was segmented as non-GM; false positive (FP), if it was a non-GM voxel in GT mask and it was segmented as GM; and finally, false negative (FN), if it was a GM voxel in GT mask and it was segmented as non-GM.

The evaluation scores included three overlapping metrics:

• Dice Similarity Coefficient (DSC): a measure of the spatial overlap between two masks (Dice, 1945).
  (1) $$\mathrm{DSC}(\mathrm{GT},\mathrm{PM})=\frac{2\times |\mathrm{GT}\cap \mathrm{PM}|}{\left|\mathrm{GT}|+|\mathrm{PM}\right|}$$
• Jaccard Index (JI): similarity index between two masks (Jaccard, 1912), which is related to the DSC.
  (2) $$\mathrm{JI}(\mathrm{GT},\mathrm{PM})=\frac{\left|\mathrm{GT}\cap \mathrm{PM}\right|}{\left|\mathrm{GT}|+|\mathrm{PM}|-|\mathrm{GT}\cap \mathrm{PM}\right|}$$
• Conformity Coefficient (CC): measures the ratio between mis-segmented voxels and correctly segmented voxels (Chang et al., 2009).
  (3) $$\mathrm{CC}(\mathrm{GT},\mathrm{PM})=\left(1-\frac{\mathrm{FP}+\mathrm{FN}}{\mathrm{TP}}\right)\times 100$$
  if the number of TP voxels is 0, the CC is undefined.

Four distance based metrics:

• Symmetric Mean Absolute Surface Distance (MSD): the mean of the sum of the Euclidean distance (for each voxel) between mask contours.
  (4) $$\mathrm{MSD}(\mathrm{GT},\mathrm{PM})=\frac{1}{N_{\mathrm{GT}}+N_{\mathrm{PM}}}\left(\overset{N_{\mathrm{GT}}}{\underset{i=1}{\sum }}|d_{i}^{\mathrm{GT}\to \mathrm{PM}}|+\overset{N_{\mathrm{PM}}}{\underset{i=1}{\sum }}|d_{i}^{\mathrm{PM}\to \mathrm{GT}}|\right)$$
  where *N*_*GT*_ and *N*_*PM*_ are the total number of voxels in the contour for GT and PM respectively. The distance values are obtained through the use of a 3D Euclidean distance transform (Gerig et al., 2001).
• Hausdorff Surface Distance (HSD): measures the maximal contour distance between the two segmentations.
  (5) $$d(X\to Y)=\max(d_{i}^{X\to Y}),i=1..N_{X}$$
  (6) HSD(GT,PM)=max(d(GT→PM),d(PM→GT))
  where *d* is the Euclidean distance between voxel *x* and *y*.
• Skeletonized Hausdorff Distance (SHD): measures the maximum distance between the two skeletonized (Zhang and Suen, 1984) GM segmentations as an indicator of maximal local error (Dupont, 2016).
• Skeletonized Median distance (SMD): measures the median distance between the two skeletonized GM segmentations as an indicator of global errors (Dupont, 2016).

And three statistical based metrics:

• Sensitivity or True Positive Rate (TPR): represents a methods ability to segment GM as a proportion of all correctly labelled voxels.
  (7) $$\mathrm{TPR}(\mathrm{GT},\mathrm{PM})=100\times \frac{\mathrm{TP}}{\mathrm{TP}+\mathrm{FN}}$$
  TPR values ranges between 0 and 100, values close to 100 mean a good quality segmentation, whilst low TPR values mean that the method tends to under-segment.
• Specificity or True Negative Rate (TNR): measures the proportion of correctly segmented background (non-GM) voxels, i.e. the ratio between the number of correctly labeled background voxels in the automated segmentation and the total number of background voxels in the manual segmentation.
  (8) $$\mathrm{TNR}(\mathrm{GT},\mathrm{PM})=100\times \frac{\mathrm{TN}}{\mathrm{TN}+\mathrm{FP}}$$
  TNR values range between 0 and 100. Methods with a lower number of FP voxels will have a higher TNR. Due to the small size of the GM when compared to the total image size, TNR values are naturally very high in this scenario.
• Precision or Positive Predictive Value, (PPV): measures the degree of compromise between true and false positive.
  (9) $$\mathrm{PPV}(\mathrm{GT},\mathrm{PM})=100\times \frac{\mathrm{TP}}{\mathrm{TP}+\mathrm{FP}}$$
  PPV values range is between 0 and 100. A high PPV (close to 100) represents optimal segmentations with a low amount or absence of FP, while low PPV values represent over-segmented results.

Skeletonized measures were calculated using the Spinal Cord Toolbox (de Leener, 2016) and the others using NiftySeg (niftyseg.sf.net). MSD, HSD, SHD and SMD are presented in millimetres, with lower scores reflecting better results. Finally, for DSC, JI, CC, TNR, TPR and PPV, higher scores reflect better results. Table 3 summarises the metrics used.

**Table 3:** A summary of the validation metrics.

**Name**	**Abbr.**	**Range**	**Qualitative Interpretation**	**Quantitative Interpretation**	**Category**
*Dice Similarity Coefficient*	*DSC*	0−1	Similarity between masks	Higher values are better	Overlap
*Jaccard Index*	*PPV*	0−100	Similarity between masks	Higher values are better	Overlap
*Conformity Coefficient*	*CC*	<100	Ratio between mis-segmented and correctly segmented	Higher values are better	Overlap
*Symmetric Mean Absolute Surface Distance*	*MSD*	>0	Mean euclidean distance between mask contours (mean error)	Smaller values are better	Distance
*Hausdorff Surface Distance*	*HSD*	>0	Longest euclidean distance between mask contours (absolute error)	Smaller values are better	Distance
*Skeletonized Hausdorff Distance*	*SHD*	>0	Indicator of maximal local error	Smaller values are better	Distance
*Skeletonized Median Distance*	*SMD*	>0	Indicator of global errors	Smaller values are better	Distance
*True Positive Rate or Sensitivity*	*TPR*	0−100	Low values mean that method tends to under-segment	Higher values are better	Statistical
*True Negative Rate or Specificity*	*TNR*	0−100	Quality of segmented background	Higher values are better	Statistical
*Positive Predictive Value or Precision*	*PPV*	0−100	Low values mean that method tends to over-segment	Higher values are better	Statistical

### Statistical analysis

Each PM was compared to the equivalent GT mask of each rater. Then, the mean and standard deviation for all the evaluation scores were computed. Both the *per* rater and overall metric results were included in a report e-mail that was sent automatically to the teams immediately after the submission of the results.

For each metric, a two-tailed unequal variance paired t-test was used to assess if the there were any significant differences in performance between the best result and the others. Tests were also performed for significant differences between each method and the consensus of the manual segmentations in order to assess the performance of the proposed techniques against human raters.

Results are presented using box plots where the bottom and the top of the box plot are the 25% and the 75% percentiles, or Q1 and Q3 quartiles, respectively; the upper and lower whiskers represent: *upper whisker=min(max(y), Q3+1.5*×*IQR)* and *lower whisker=max(min(y), Q1-1.5*×*IQR)*, where *IQR* stands for interquartile range that is the difference between Q3 and Q1. Additionally, each of the obtained results is represented as a black dot and the mean using a rhombus.

Using STATA 14, we computed a generalized linear model to assess whether the results of any presented method and metric were biased by sequence or age. All sequence interaction coefficients (categorical variables) were jointly compared with an F-test to estimate between-sequence differences. Interactions with age (continuous variable) were obtained using an independent linear regression model without sequence interaction.

### Submission guidelines

In this challenge, the participating teams were allowed unlimited submissions. Teams were also allowed to use other publicly available datasets within their algorithms. Numerical input parameters were permitted, but under the requirement that they would be kept constant for all data sets. Output GM segmentations were provided in the same space and resolution as the input data. There were no restrictions on how the algorithms were implemented with regards to platform, programming language, or software library dependencies. Algorithms were executed solely by the competing team with the segmentation results provided to the organizers. Output segmentations were saved in NIFTI format with a label of 1 assigned to spinal cord GM and 0 otherwise. Methods and results were presented during the 3rd Annual Spinal Cord MRI Workshop, held immediately following the ISMRM annual meeting in Singapore, May 2016. If human interaction was required to run an algorithm, teams were asked to provide a description of the required steps (e.g., cropping, normalization, centering, pre-segmentation, etc.).

## Methods

Eleven different users requested the data and seven institutions initially entered the challenge. Finally, six teams submitted final results to the challenge and presented their method during the workshop.

• Team 1 – University College London, led by FP, MJC, CWK and SO. Method name: *Joint collaboration for spinal cord grey matter segmentation* (Prados et al., 2016b), referred to as: JCSCS.
• Team 2 – University of British Columbia, led by EL, TB and RT. Method name: *Deepseg*, referred to as: DEEPSEG.
• Team 3 – University of California San Francisco, led by ED, NP, RS, AZ, JCG and RH. Method name: *Morphological geodesic active contours algorithm* (Datta et al., 2016) -, referred to as: MGAC.
• Team 4– Eindhoven University of Technology and University College London, led by SS, FG, FP and CWK. Method name: *Grey matter segmentation based on maximum entropy*, referred to as: GSBME.
• Team 5 – Polytechnique Montreal, led by SMD, BDL and JCA. Method name: *Multi-atlas based segmentation method for the spinal cord white and grey matter* (Dupont, 2016) implemented in the Spinal Cord Toolbox (de Leener, 2016), referred to as: SCT.
• Team 6 – University of Zurich and University College London, led by CB, PF and JA. Method name: *Semisupervised VBEM* (Blaiotta et al., 2016), referred to as: VBEM.

All methods are described in the Appendix A and Table 4 presents a summary of each method. No modifications were introduced to any of the presented spinal cord GM segmentation methods as a result of this challenge for the purposes of this publication.

**Table 4:** Setup parameters and characteristics for each presented method. Note atlas size is in number of slices and that computational time per slice is an approximation, has been obtained in different workstations and might vary depending on the resolution.

**Name**	**Init.**	**Training**	**Atlas size**	**Time per slice**	**Available**
**JCSCS**	Automatic	No	820	4–5 min	niftyseg.sf.net
**DEEPSEG**	Automatic	Yes (4 h)	160	<1 s	*Soon*
**MGAC**	Automatic	No	1	1 s	*Soon*
**GSBME**	Manual	Yes (<1 min)	No	5–80 s	*Upon request*
**SCT**	Automatic	No	447	8–10 s	spinalcordtoolbox.sf.net
**VBEM**	Automatic	No	No	5 s	*Soon*

## Results

**Fig. 1:**
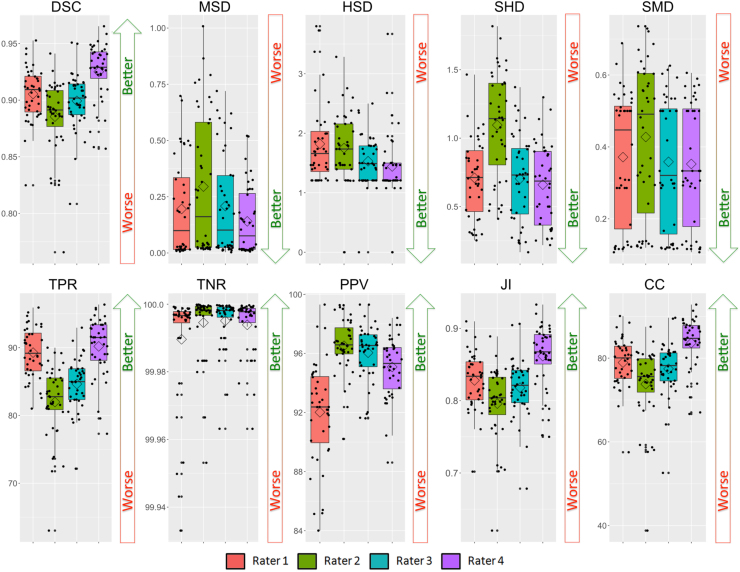
Results of the raters for the testing dataset. Boxplot, the mean value is represented by a rhombus and dots show original obtained values per mask. Each rater's results are compared to the majority voting mask. From left to right, first row: Dice similarity coefficient (DSC), mean surface distance (MSD), Hausdorff surface distance (HSD), skeletonized median distance (SMD) and skeletonized Hausdorff distance (SHD). Second row: true positive rate (TPR), true negative rate (TNR), positive predictive value (PPV), Jaccard index (JI) and conformity coefficient (CC).

In order to assess inter-rater variability, using leave-one-out cross-validation, the quantitative analysis results of the performance of each rater segmentation using the testing dataset are presented in Table 5 and Fig. 1.

**Table 5:** Comparison of each rater segmentation versus the majority voting mask of all raters for the test dataset with the mean (std) Dice similarity coefficient (DSC), mean surface distance (MSD), Hausdorff surface distance (HSD), skeletonized Hausdorff distance (SHD), skeletonized median distance (SMD), true positive rate (TPR), true negative rate (TNR), positive predictive value (PPV), Jaccard index (JI) and conformity coefficient (CC). In bold face, the best obtained result for each particular metric. The script * represents significant differences (paired t-test with p<0.05) between the obtained result by a rater and the best result. MSD, HSD, SHD and SMD are in millimetres and lower values mean better, for all the other scores higher values mean better score.

	**Rater 1**	**Rater 2**	**Rater 3**	**Rater 4**
*DSC*	0.91 (0.02)*	0.89 (0.03)*	0.90 (0.03)*	0.93 (0.03)
*MSD*	0.20 (0.21)	0.30 (0.31)*	0.21 (0.22)	0.14 (0.15)
*HSD*	1.80 (0.68)*	1.75 (0.57)*	1.53 (0.44)	1.44 (0.55)
*SHD*	0.71 (0.28)	1.10 (0.39)*	0.70 (0.31)	0.66 (0.30)
*SMD*	0.37 (0.18)	0.43 (0.21)	0.36 (0.18)	0.35 (0.17)
*TPR*	89.27 (3.7)	81.99 (5.39)*	84.64 (3.76)*	90.19 (4.38)
*TNR*	99.990 (0.02)	99.995 (0.01)	99.995 (0.01)	99.994 (0.01)
*PPV*	92.01 (3.48)*	96.52 (1.87)	96.04 (1.92)	95.08 (2.06)*
*JI*	0.83 (0.04)*	0.80 (0.05)*	0.82 (0.04)*	0.86 (0.04)
*CC*	78.95 (5.94)*	73.80 (8.89)*	77.45 (6.40)*	83.62 (6.21)

**Fig. 2:**
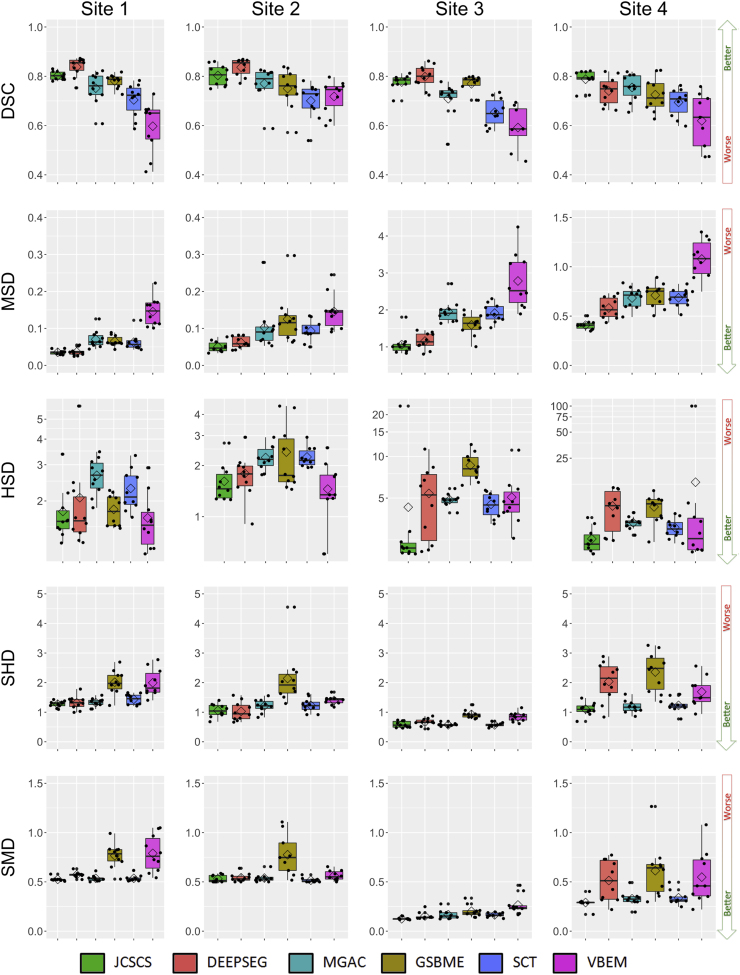
Dice similarity coefficient (DSC), mean surface distance (MSD), Hausdorff surface distance (HSD), skeletonized Hausdorff distance (SHD), skeletonized median distance (SMD) results of the presented methods per site using the testing dataset. Boxplot, the mean value is represented by a rhombus and dots show original obtained values per mask. MSD, HSD, SMD and SHD are in mm and represented using a logarithmic scale.

**Fig. 3:**
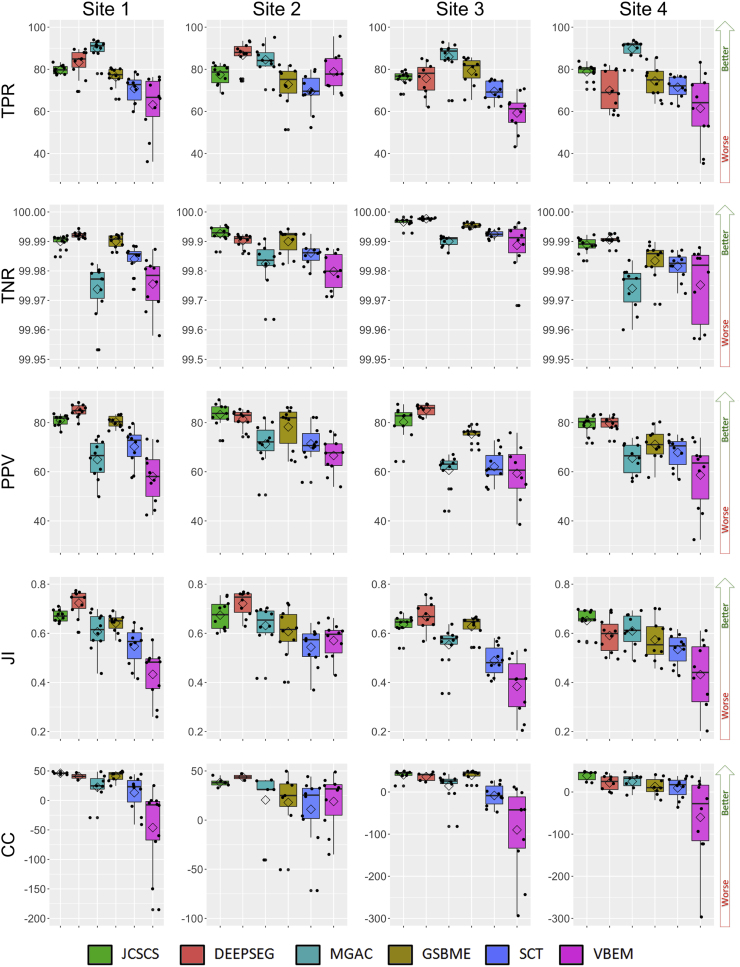
True positive rate (TPR), true negative rate (TNR), positive predictive value (PPV), Jaccard index (JI) and conformity coefficient (CC) results of the presented methods per site using the testing dataset. Boxplot, the mean value is represented by a rhombus and dots show original obtained values per mask.

In addition, Table 6, Figs. 2 and 3 present the results of each method also using the testing dataset against each rater independently. In Figs. 2 and 3, the results are split by site, with a boxplot drawn for each metric and site. In Fig. 2, MSD, HSD, SMD and SHD are in millimetres but are represented using a logarithmic scale in order to highlight the various results. Table 6 presents the obtained results per method, with the mean (std) and p-value for each metric estimated with respect to the best result of the same metric (marked in bold face). Using a two-tailed unequal variance paired t-test, significant differences (p<0.05) between a method and the best performing method have been marked with “*”. Methods that were found to not be statistically significantly different from the consensus of manual segmentations are marked with script “+” (p>0.05).

**Table 6:** Comparison of each method segmentation versus each one of the four raters masks for the test dataset with the mean (std) Dice similarity coefficient (DSC), mean surface distance (MSD), Hausdorff surface distance (HSD), skeletonized Hausdorff distance (SHD), skeletonized median distance (SMD), true positive rate (TPR), true negative rate (TNR), positive predictive value (PPV), Jaccard index (JI) and conformity coefficient (CC). In bold face, the best obtained result for each particular metric. The script * represents significant differences (paired t-test with p<0.05) between the obtained result and the best result. The script + represents non-significant differences (paired t-test with p>0.05) between the obtained result and the consensus of the raters. MSD, HSD, SHD and SMD are in millimetres and lower values mean better, for all the other scores higher values mean better score.

	**JCSCS**	**DEEPSEG**	**MGAC**	**GSBME**	**SCT**	**VBEM**
*DSC*	0.79 (0.04)	0.80 (0.06)	0.75 (0.07)*	0.76 (0.06)*	0.69 (0.07)*	0.61 (0.13)*
*MSD*	0.39 (0.44)	0.46 (0.48)	0.70 (0.79)*	0.62 (0.64)	0.69 (0.76)*	1.04 (1.14)*
*HSD*	2.65 (3.40)+	4.07 (3.27)*	3.56 (1.34)	4.92 (3.30)*	3.26 (1.35)	5.34 (15.35)+
*SHD*	1.00 (0.35)	1.26 (0.65)*	1.07 (0.37)	1.86 (0.85)*	1.12 (0.41)	2.77 (8.10)+
*SMD*	0.37 (0.18)+	0.45 (0.20)*+	0.39 (0.17)*+	0.61 (0.35)*	0.39 (0.16)+	0.54 (0.25)*
*TPR*	77.98 (4.88)*	78.89 (10.33)*	87.51 (6.65)+	75.69 (8.08)*	70.29 (6.76)*	65.66 (14.39)*
*TNR*	99.98 (0.03)	99.97 (0.04)	99.94 (0.08)*	99.97 (0.05)	99.95 (0.06)	99.93 (0.09)*
*PPV*	81.06 (5.97)	82.78 (5.19)	65.60 (9.01)*	76.26 (7.41)*	67.87 (8.62)*	59.07 (13.69)*
*JI*	0.66 (0.05)	0.68 (0.08)	0.60 (0.08)*	0.61 (0.08)*	0.53 (0.08)*	0.45 (0.13)*
*CC*	47.17 (11.87)	49.52 (20.29)	29.36 (29.53)*	33.69 (24.23)*	6.46 (30.59)*	−44.25 (90.61)*

**Fig. 4:**
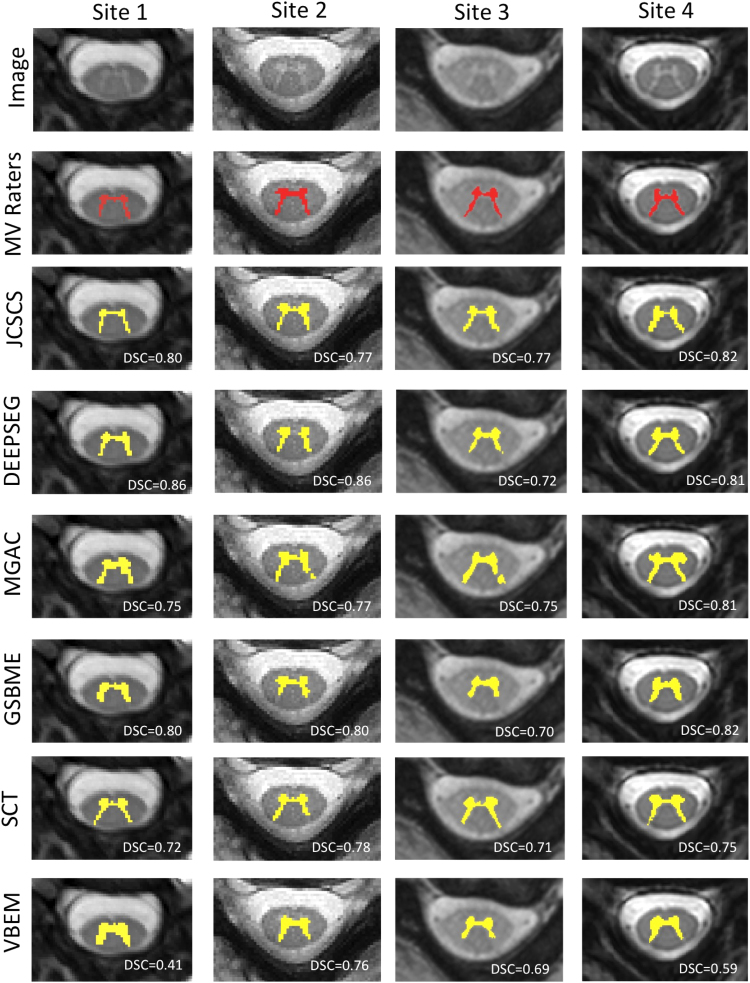
Binary grey matter segmentation results for the same single slice for subject 11 of each site. From top to bottom row: input image, majority voting segmentation from the 4 raters and the segmentation methods: JCSCS, DEEPSEG, MGAC, GSBME, SCT and VBEM. Obtained 3D DSC is overlayed.

For a qualitative analysis, a randomly selected slice is shown from subject 11 of each site. Original image, consensus segmentation mask from the four raters, the corresponding binary segmentation result for each method and DSC value are shown (see Fig. 4).

Using the WM and GM consensus masks, we computed the mean and standard deviation of ${\mathrm{SNR}}_{\mathrm{WM}}$ and CNR. Site 1 had a ${\mathrm{SNR}}_{\mathrm{WM}}=11.01\pm 1.28$ and a CNR=0.85±0.27. Site 2 had a ${\mathrm{SNR}}_{\mathrm{WM}}=9.65\pm 1.60$ and a CNR=1.19±0.15. Site 3 had a ${\mathrm{SNR}}_{\mathrm{WM}}=7.06\pm 1.72$ and a CNR=0.66±0.14. Finally, Site 4 had a ${\mathrm{SNR}}_{\mathrm{WM}}=8.36\pm 1.30$ and a CNR=0.92±0.13.

The generalized linear model for assessing bias related to the type of sequence (see Table 7) showed that DEEPSEG results are significantly affected (p<0.05) by image quality (i.e. site) for all the metrics. We also found that most of the distance metrics results obtained by the methods (MSD, HSD, SHD and SMD) are influenced by the sequences (p<0.05) due to the different resolutions. Furthermore, Table 8 shows that age (atrophy) significantly influences the JCSCS and GBSME algorithms when overlap metrics are considered (DSC, JI and CC).

**Table 7:** Generalized linear model results for the method's performance per each metric depending on the scanner sequence expressed as p-value (F-test between all site coefficients in a regression model). Values with p<0.05 (in bold face) mean that the image quality has an statistically significant influence over the performance of this metric and method. Dice similarity coefficient (DSC), mean surface distance (MSD), Hausdorff surface distance (HSD), skeletonized Hausdorff distance (SHD), skeletonized median distance (SMD), true positive rate (TPR), true negative rate (TNR), positive predictive value (PPV), Jaccard index (JI) and conformity coefficient (CC).

	**JCSCS**	**DEEPSEG**	**MGAC**	**GSBME**	**SCT**	**VBEM**
*DSC*	0.233	<**0.001**	0.210	0.174	0.286	**0.010**
*MSD*	<**0.001**	<**0.001**	<**0.001**	<**0.001**	<**0.001**	<**0.001**
*HSD*	0.270	<**0.001**	<**0.001**	<**0.001**	<**0.001**	0.295
*SHD*	<**0.001**	<**0.001**	<**0.001**	<**0.001**	<**0.001**	0.345
*SMD*	<**0.001**	<**0.001**	<**0.001**	<**0.001**	<**0.001**	<**0.001**
*TPR*	0.120	<**0.001**	0.145	0.263	0.869	**0.005**
*TNR*	<**0.001**	<**0.001**	<**0.001**	<**0.001**	<**0.001**	<**0.001**
*PPV*	0.155	<**0.001**	**0.032**	**0.009**	**0.030**	0.140
*JI*	0.217	<**0.001**	0.172	0.212	0.261	**0.003**
*CC*	0.256	<**0.001**	0.289	0.161	0.346	**0.041**

**Table 8:** Generalized linear model results for the method's performance per each metric depending on the age of each subject expressed as regression coefficient, 95% confidence interval (CI) and p-value. DSC, TPR, TNR, PPV, JI, CC are in ${\mathrm{years}}^{-1}$ and SHD, SMD, MSD, HSD are in mm years^−1^. Values with p<0.05 mean that the age (atrophy) has a statistically significant influence over the performance of this metric and method. Dice similarity coefficient (DSC), mean surface distance (MSD), Hausdorff surface distance (HSD), skeletonized Hausdorff distance (SHD), skeletonized median distance (SMD), true positive rate (TPR), true negative rate (TNR), positive predictive value (PPV), Jaccard index (JI) and conformity coefficient (CC).

	**JCSCS**	**DEEPSEG**	**MGAC**	**GSBME**	**SCT**	**VBEM**
*DSC*	$9\times 10^{-}4$	0.001	−0.001	0.001	$-3\times 10^{-}4$	$9\times 10^{-}4$
	CI=[$1\times 10^{-}4$ to $17\times 10^{-}4$]	CI=[$-2\times 10^{-}4$ to $26\times 10^{-}4$]	CI=[$-27\times 10^{-}4$ to $6\times 10^{-}4$]	CI=[$-1\times 10^{-}4$ to $30\times 10^{-}4$]	CI=[$-19\times 10^{-}4$ to $13\times 10^{-}4$]	CI=[$-41\times 10^{-}4$ to $21\times 10^{-}4$]
	*p***=0.02**	*p*=0.11	*p*=0.22	*p***=0.03**	*p*=0.70	*p*=0.54
						
*MSD*	−0.002	−0.003	$2\times 10^{-}4$	−0.005	−0.002	$7\times 10^{-}4$
	CI=[−0.013 to 0.008]	CI=[−0.014 to 0.009]	CI=[−0.020 to 0.020]	CI=[−0.021 to 0.011]	CI=[−0.021 to 0.018]	CI=[−0.028 to 0.030]
	*p*=0.66	*p*=0.65	*p*=0.99	*p*=0.53	*p*=0.87	*p*=0.96
						
*HSD*	−0.036	−0.022	−0.013	−0.049	−0.009	0.070
	CI=[−0.121 to 0.049]	CI=[−0.104 to 0.059]	CI=[−0.045 to 0.018]	CI=[−0.130 to 0.034]	CI=[−0.040 to 0.023]	CI=[−0.321 to 0.460]
	*p*=0.40	*p*=0.58	*p*=0.40	*p*=0.24	*p*=0.58	*p*=0.72
						
*SHD*	−0.002	−0.012	$5\times 10^{-}4$	−0.021	$5\times 10^{-}4$	0.043
	CI=[−0.009 to 0.005]	CI=[−0.028 to 0.003]	CI=[−0.008 to 0.009]	CI=[−0.040 to −0.002]	CI=[−0.009 to 0.010]	CI=[−0.163 to 0.249]
	*p*=0.62	*p*=0.12	*p*=0.90	*p***=0.03**	*p*=0.91	*p*=0.67
						
*SMD*	0.001	$-8\times 10^{-}4$	$-7\times 10^{-}4$	−0.004	$8\times 10^{-}4$	0.003
	CI=[−0.003 to 0.005]	CI=[−0.006 to 0.004]	CI=[−0.003 to 0.005]	CI=[−0.012 to 0.004]	CI=[−0.003 to 0.005]	CI=[−0.003 to 0.009]
	*p*=0.63	*p*=0.75	*p*=0.72	*p*=0.31	*p*=0.65	*p*=0.32
						
*TPR*	0.074	0.085	−0.097	0.102	−0.052	−0.199
	CI=[−0.019 to 0.167]	CI=[−0.165 to 0.335]	CI=[−0.254 to 0.059]	CI=[−0.084 to 0.288]	CI=[−0.213 to 0.110]	CI=[−0.554 to 0.155]
	*p*=0.11	*p*=0.49	*p*=0.21	*p*=0.27	*p*=0.52	*p*=0.26
						
*TNR*	$6\times 10^{-}4$	$7\times 10^{-}4$	$0.5\times 10^{-}4$	$8\times 10^{-}4$	$-4\times 10^{-}4$	$12\times 10^{-}4$
	CI=[$-2\times 10^{-}4$ to $14\times 10^{-}4$]	CI=[$-3\times 10^{-}4$ to $17\times 10^{-}4$]	CI=[$-19\times 10^{-}4$ to $20\times 10^{-}4$]	CI=[$-2\times 10^{-}4$ to $20\times 10^{-}4$]	CI=[$-10\times 10^{-}4$ to $19\times 10^{-}4$]	CI=[$-9\times 10^{-}4$ to $33\times 10^{-}4$]
	*p*=0.13	*p*=0.19	*p*=0.96	*p*=0.13	*p*=0.57	*p*=0.24
						
*PPV*	0.109	0.125	−0.099	0.221	−0.013	−0.006
	CI=[−0.010 to 0.229]	CI=[0.039–0.211]	CI=[−0.307 to 0.108]	CI=[0.068–0.373]	CI=[−0.210 to 0.183]	CI=[−0.344 to 0.331]
	*p*=0.07	*p***=0.005**	*p*=0.34	*p***=0.006**	*p*=0.89	*p*=0.97
						
*JI*	0.001	0.002	−0.001	0.221	$3\times 10^{-}4$	−0.001
	CI=[$2\times 10^{-}4$ to $24\times 10^{-}4$]	CI=[$-4\times 10^{-}4$ to $35\times 10^{-}4$]	CI=[$-32\times 10^{-}4$ to $8\times 10^{-}4$]	CI=[$2\times 10^{-}4$ to $37\times 10^{-}4$]	CI=[$-22\times 10^{-}4$ to $14\times 10^{-}4$]	CI=[−0.004 to 0.002]
	*p***=0.02**	*p*=0.12	*p*=0.24	*p***=0.03**	*p*=0.68	*p*=0.46
						
*CC*	0.312	0.400	−0.461	0.623	0.104	−0.447
	CI=[0.049–0.574]	CI=[−0.081 to 0.883]	CI=[−1.170 to 0.249]	CI=[0.068–1.179]	CI=[−0.845 to 0.638]	CI=[−2.704 to 1.810]
	*p***=0.02**	*p*=0.10	*p*=0.20	*p***=0.03**	*p*=0.78	*p*=0.69

## Discussion

Presented algorithms were found to be able to identify and segment GM on all datasets with an acceptable precision and shape (see Fig. 4). It is important to highlight the fact that the small size of the spinal cord GM makes the process of segmenting the GM algorithmically challenging, as the inclusion/exclusion of one voxel can have a substantial impact on the performance scores (see Table 7, Table 8).

Raters delineated very similar masks, however in comparison to the majority voting-based consensus segmentation, rater 4 performs significantly better than the remaining raters (see Table 5). Note that significant differences in performance between raters does not necessarily mean clinically significant differences. The statistically significant difference between raters is mostly due to small differences in segmentation protocol when drawing the GM. This is further corroborated by the small standard deviations of most performance scores (see Table 5 and Fig. 1).

JCSCS was found to be a method that provides similar mask contour and shape when compared to the ground truth, obtaining amongst some of the best scores for MSD, HSD, SHD and SMD (see Table 6 and Fig. 2). In terms of HSD and SMD, JCSCS was not found to be significantly different from a consensus manual rater (p>0.05; see Table 6). Regarding the overlap scores between JCSCS and rater masks, the obtained results were found not to differ significantly (p>0.05) from the best results (see DSC, PPV, JI and CC in Table 6). The low TPR values obtained by JCSCS means that it tends to marginally undersegment the GM producing more conservative masks and consequently getting high TNR values. The lowest standard deviation obtained by JCSCS in seven out of ten validation metrics demonstrates its robustness and reliability across different vendors, independent sites, various acquisition parameters and image resolution.

With the highest DSC among all presented techniques (DSC=0.8) DEEPSEG has shown the potential of deep learning for spinal cord segmentation. Furthermore, the algorithm, which was originally intended for brain lesion segmentation, was only slightly adjusted for the spinal cord, lending further support to the strengths of deep learning. The DEEPSEG algorithm performs significantly worse than the best technique on four scores: HSD, SHD, SMD, and TPR. However, the SMD, which quantifies global errors, was found not to be significantly different from human raters, suggesting that DEEPSEG captures the gold standard skeletonised structure of the GM. In some occurrences, the DEEPSEG algorithm will fail to connect the two horns of the GM, as seen in Fig. 4, potentially linked to the relatively low number of training samples commonly necessary for deep learning applications.

The MGAC method scored high amongst the methods in TPR, indicating the highest level of specificity. In addition, the MGAC method scored amongst the highest in both SHD and SMD, demonstrating the methods ability to determine the underlying shape of the GM. However, MGAC did not score as highly in TNR, representing a lower level of sensitivity. This lower sensitivity is also seen in the lower MSD and PPV scores. These results suggest that the MGAC method is excellent at determining the underlying shape of the GM, but may overestimate the GM volumes compared to human raters. This overestimation in volume can be seen in Fig. 4. One strong advantage of the MGAC algorithm is its ability to work on images with different contrasts. This algorithm was developed for use on PSIR images, but has also been shown to work well on T2${}^{*}$weighted images.

The GSBME method consists of three steps: preprocessing, maximum-entropy thresholding and outlier detection. As far as its performance is concerned, GSBME provides consistent values of quality-of-segmentation scores in all sites. It ranks intermediately when scores that measure the degree of overlap between masks (i.e. DSC and JI) or the ability of rejecting false/accepting true segmentations are considered (i.e. TNR and TPR). However, its performance worsens when using scores that measure the physical distance between segmented voxels, especially in their skeletonized version (i.e. SMD, SHD). While the performance of the algorithm could be potentially improved, the current implementation appears to suffice for the characterisation of grey/white matter differences in future studies involving healthy controls. From an algorithmic point of view, the current implementation includes a number of operations to standardise data from different sites (i.e. normalisation, denoising). These could potentially be unnecessary for single-site studies, leading to a further simplification of the algorithm. The bottle-neck of the method is the initial detection of the spinal cord, a semi-automatic procedure requiring manual input. Further improvements to the technique should focus on the final step of outlier detection, which effectively reduces false positives but that can also lead to false negatives.

The GM segmentation as implemented in the Spinal Cord Toolbox (SCT) is an atlas-based method. Therefore, the output segmentation is a fusion of manual segmentations that constitute the model, implying that segmentations always have a shape that resembles the GM. Moreover, unlike contour deformation or intensity based methods, SCT is very robust to artefacts or pathologies as demonstrated in Dupont (2016). The scores computed for SCT were satisfying. However, the relatively low PPV suggests that SCT has a tendency to over segment the GM, which could be addressed by adjusting the threshold of the output probabilistic segmentation. Results obtained with SCT for shape sensitive indicators (HSD, SHD and SMD) were amongst the best ones, suggesting that SCT captures properly the GM shape in the input image. Moreover, the SMD score of SCT was similar to the gold standard, suggesting that this method performs as well as human raters in capturing the overall GM shape. Finally, SCT is available as an open-source software package (http://spinalcordtoolbox.sf.net) (de Leener, 2016).

Compared to some of the other competing algorithms, the semisupervised VBEM method exhibited a relatively poor performance in terms of overlap scores with the manual segmentations. On the other hand, the results were submitted for evaluation only once. Therefore no parameter tuning was performed in order to maximize the performance with respect to the selected accuracy measures. The method tries to capture the most parsimonious partitioning of the data based on the observed image intensities, therefore structures that have partially overlapping intensity distributions, such as GM and WM in the spinal cord might be particularly hard to resolve. Additionally, if the training data set is not sufficiently large, volumetric approaches also suffer from having a relatively small amount of training labels available at each anatomical cross section (especially for images with different fields of view), compared to slice based methods. Nevertheless, such a probabilistic modelling framework represents an ideal environment for performing statistical morphometric group studies, which can potentially help to unravel the mechanisms underlying neurological disorders. It should also be noted that, for this purpose, conformity with manual labelling protocols does not constitute a primary concern, as long as there is internal consistency of the results across subjects.

Finally, as no single method has consistently outperformed all other methods for every site and assessment metric, no hard conclusions can be drawn with regards to the true best performing method; the choice of an optimal method would change depending on the target sequence, computational time and choice of performance metrics.

## Conclusions

This paper demonstrates the feasibility of six emerging segmentation methods to fully automatically and robustly segment the butterfly shape of the GM in the spinal cord. Thus, next to established voxel-wise segmentation algorithms optimized for the brain, the spinal cord tissue is entering the field of voxel-wise analysis opening new avenues to make statistical inferences of volume and shape across the entire neuroaxis (Freund et al., 2016).

We have presented the results of the first spinal cord GM segmentation challenge. Six institutions across the world have collaborated in order to compare their cutting edge methods using the same dataset from multiple vendors and sites. The challenge was successful and the presented methods provided highly promising results using different underlying principles. This variety showed that spinal cord GM segmentation remains challenging within a vibrant research field.

Finally, training data and masks, and testing data without masks, will remain publicly available at http://cmictig.cs.ucl.ac.uk/niftyweb for the community to continue to evaluate their methods.

Future spinal cord GM challenges will aim to include other image modalities, more vendors, neurological conditions, other spinal cord levels and attempt to harmonise the manual segmentation software and protocol.

## Figures and Tables

**Fig. 5:**
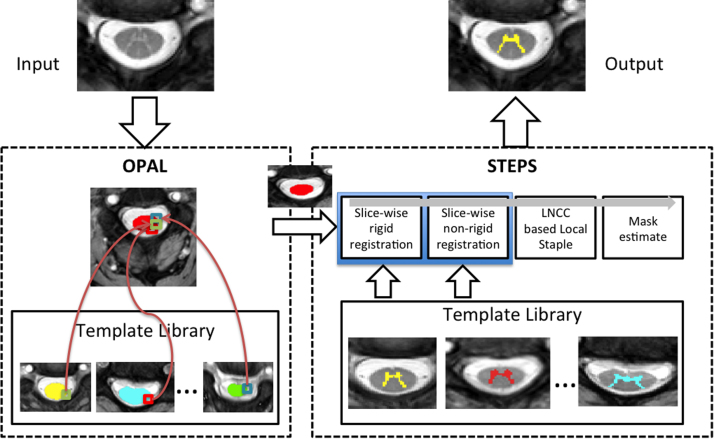
Schematic representation of the proposed pipeline.

**Fig. 6:**
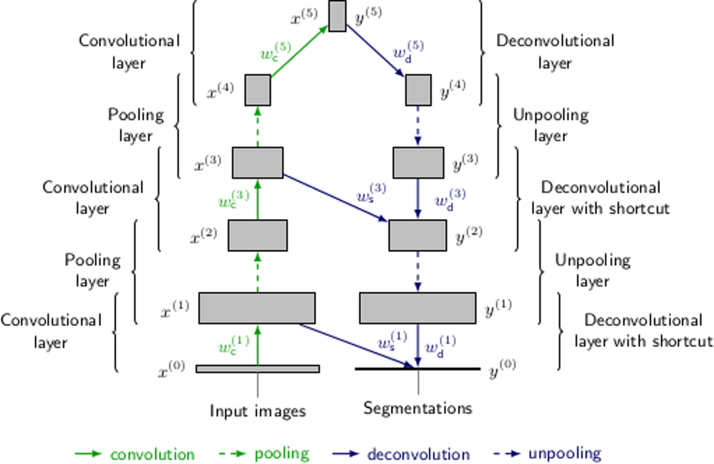
Diagram showing the 11-layer network structure used in the present work, based on the network presented by Brosch et al. (2016). The shortcut connections between corresponding convolutional and deconvolutional layers allow for the learning of features at different scales.

**Fig. 7:**
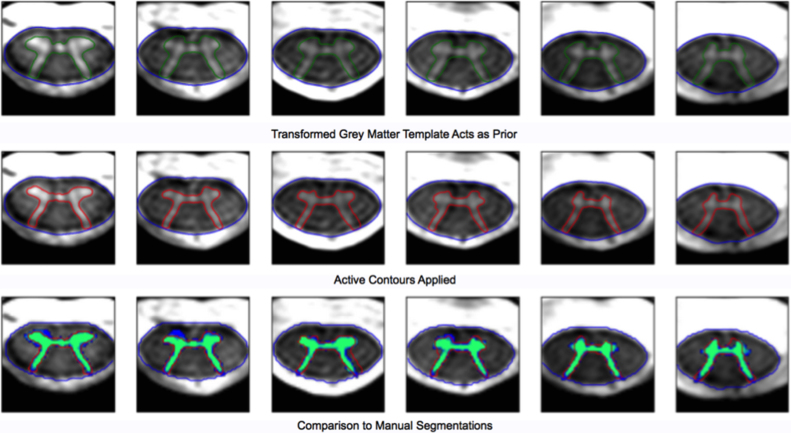
Example of MGAC with comparisons to manual segmentations.

**Fig. 8:**
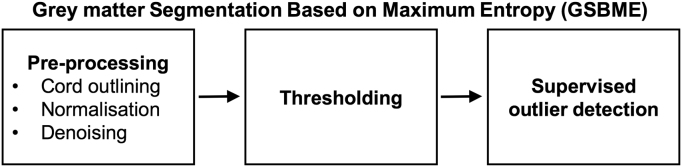
Block diagram describing the three-stage GSBME procedure for grey matter segmentation in anatomical MRI data of the spinal cord. The first step of the procedure is pre-processing, which implements whole-cord segmentation, signal intensity normalisation and image denoising. The second step is the thresholding of the sum of grey/white matter signal intensity entropies. The final stage consists of a one-class classifier for supervised outlier detection.

**Fig. 9:**
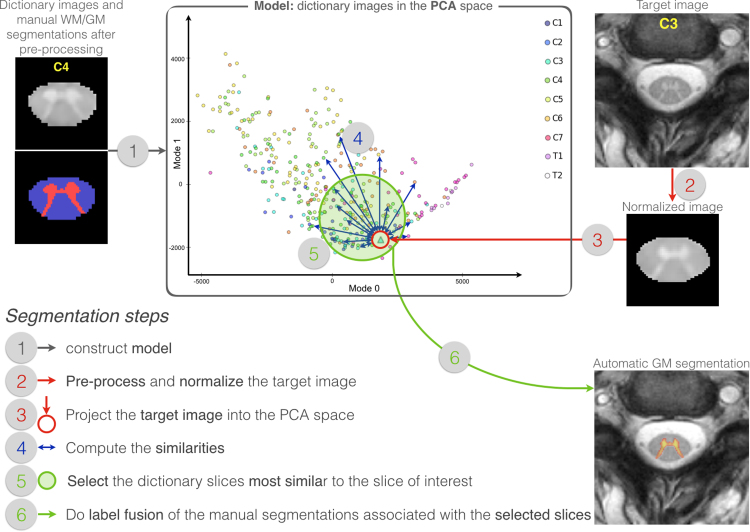
Multi-atlas based segmentation method.

**Fig. 10:**
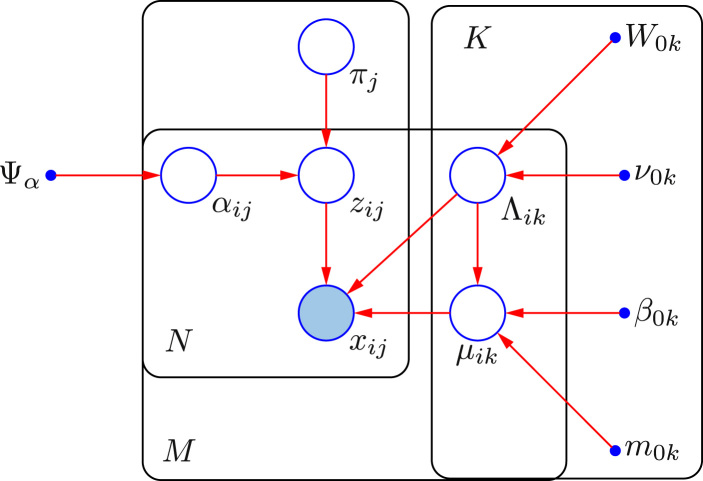
Directed acyclic graph representing the Gaussian mixture model that the VBEM method relies on.

## Joint collaboration for spinal cord grey matter segmentation

The proposed method (Prados et al., 2016b) combines two existing label fusion segmentation techniques: OPAL (Optimized PatchMatch Label Fusion) (Ta et al., 2014, Prados et al., 2015, Giraud et al., 2016) for detecting the spinal cord and STEPS (Similarity and Truth Estimation for Propagated Segmentations) (Cardoso et al., 2013) to accurately segment the GM. The proposed method uses a multi-atlas segmentation propagation strategy with all registrations and segmentations performed in a 2D slice-wise manner before merging them into a 3D volume. A schematic representation of the proposed pipeline is shown in Fig. 5.

Due to the low computational time and decent segmentation accuracy in some applications, OPAL is used here in its original form to simply localise the spinal cord. This cord localisation step is achieved by providing a dictionary of spinal cord images and associated manually segmented cords to the OPAL algorithm, all of which are then propagated to the new unseen image. This step has an average computational time of less than 1 s. The rough cord localisation obtained from OPAL is then used to initialise a multi-atlas propagation approach.

The main characteristic of STEPS is that it introduces a spatially variant image similarity term the classical STAPLE framework (Cardoso et al., 2013), enabling the characterisation of both image similarity and human rater performance in a unified manner. The STEPS segmentation process is divided in two stages: segmentation propagation and fusion. Starting from a template library with associated manual segmentations, all the templates are first registered to the target image using initially a rigid-only registration and then a non-rigid registration. All registrations were done using the NiftyReg software package (niftyreg.sf.net). The normalised cross correlation (NCC) is then estimated between each deformed template and the target image, quantifying the similarity between the two images. The top *X* most similar deformed templates according to the NCC are then finally fused into a consensus segmentation using STEPS. STEPS uses the locally normalised cross correlation (LNCC) between the registered template images and the target image to locally select the best atlases to fuse. A consensus probabilistic GM segmentation is obtained using the STEPS algorithm as implemented in NiftySeg (niftyseg.sf.net). The probabilistic nature of the consensus segmentation implicitly encodes partial volume effect, improving tissue boundary localisation and delineation. Finally, the probabilistic consensus masks are thresholded at 0.5 to produce binary segmentations.

Note that, in order to increase the performance of the fusion step, the centre of mass of the OPAL cord segmentation is used to initialise the rigid registration step between templates and target image. The OPAL cord segmentation is also used to mask non-cord regions from the non-linear registration step, further improving the performance of the template registration step.

All experiments used the following parameters. OPAL was used with the original parameters (Ta et al., 2014, Giraud et al., 2016): the 2D patch size was 5x5 and the number of inner iterations was 5. Finally, the numbers of threads and the number of best-matches were both set to 10. STEPS also used the original parameters (Cardoso et al., 2013): the number of best templates was *X*=15, standard deviation of the Gaussian smoothing kernel for LNCC estimation σ=1.5 and the Markov Random Field (MRF) spatial consistency set to 0.55. In order to maximise the size of the STEPS library, all the scans were left-right flipped.

Both, OPAL and STEPS, are public available inside NiftySeg software package (niftyseg.sf.net), thus making the method fully open source.

## Deepseg

The implementation of Deepseg is a further development of the deep 3D convolutional encoder network with shortcut connections proposed by Brosch et al. (2016). The neural network (LeCun et al., 1998) used in that study was optimized for lesion segmentation in the brain in subjects with multiple sclerosis (MS). Since the neural network approach does not make any assumptions that are specific to the segmentation of MS lesions, the same approach can be used for a variety of segmentation problems such as the segmentation of GM in the spinal cord.

The network structure used in this work is similar to the u-net (Ronneberger et al., 2015) structure with a contracting and an expanding pathway. The contracting pathway consists of alternating convolutional (Lecun et al., 1998) and pooling layers. The expanding pathway consists of alternating deconvolutional (Zeiler et al., 2011) and unpooling layers, in contrast to the u-net structure which consists of alternating convolutional and upsampling operations. This allows the Deepseg network structure to produce feature maps of exactly the same size as the corresponding convolutional layers, which enables easy shortcut connections between layers (Brosch et al., 2016). This is utilized in the Deepseg algorithm to directly predict the entire segmentation without special handling of the border region, see Fig. 6.

To optimize the method for GM segmentation, the network design proposed by Brosch et al. (2016) was slightly adjusted. Each pathway of the network was extended with two more layers, extending the model from 7 to 11 layers. This ensures that the receptive field of the neurons captures the full size of the spinal cord. As a consequence of this, the pre-training step outlined in Brosch et al. (2016) that is used to obtain initial parameters for the convolutional layers was modified to include 3 convolutional restricted Boltzmann machines (Lee et al., 2011) to provide prediction parameters to all the layers of the fine-tuning network shown in Fig. 6.

Instead of using the commonly used sum of squared differences or cross-entropy as the objective function, a weighted sum of two terms: the mean square differences of the GM voxels and the non-GM voxels (Brosch et al., 2016) was used. The weighting of these terms will balance the sensitivity (the first term) and specificity (second term) of the final segmentation. The sensitivity threshold was fixed at 0.1 in this study.

Before training the model, all scans were resampled to an in-plane voxel size of 0.25×0.25 mm^2^ and subsequently cropped to a standardized image size of 256×256 in-plane with 12 slices. If the volume contained more than 12 slices, the central 12 slices were chosen, and, if the volume had fewer than 12 slices, zero-padding was performed. Image cropping was only performed on the training images to reduce the training time. When applied to new images, the network can be resized to match the size of new images.

The same network structure was used to train two models; one for full cord segmentation, and another for GM segmentation. In the first step, the full cord is segmented, and the image is subsequently cropped to a region of interest of size 100×100 voxels, with the cord centered. In the second step, the cropped cord is used to predict a probabilistic GM segmentation, which is then binarized using a constant threshold. The threshold is empirically tuned as part of the training procedure and the same threshold is used to produce all subsequent segmentations. The binary GM segmentation is then warped back to native image space. With data from 40 subjects and manual GM segmentations from 4 raters, a total of 160 training pairs were used for training the model for the GM segmentation. Training of the entire network took 4 h using a NVIDIA GeForce GTX 660 with 960 cores operating at 1.03 GHz using a custom GPU implementation developed by Brosch and Tam (2015). Prediction, i.e. segmentation, of all the data took only a few seconds using the same hardware.

## Morphological geodesic active contours algorithm

This spinal cord GM segmentation technique (Datta et al., 2016) uses shape template registration methods along with Morphological Geodesic Active Contour (MGAC) models.

### Preprocessing

In each of the images from the training and test set, the whole spinal cord was first segmented using the software JIM (v. 6.0, Xinapse Systems, Northants, UK; http://www.xinapse.com/). Then, all images were up-sampled and cropped so that the resulting images were centered on the segmented spinal cord and each slice had a field of view of 15 mm×15 mm and a resolution of 0.05 mm×0.05 mm.

### Creating level specific templates

Level specific templates of the GM and the whole cord were first created from the training set. Distance maps were created from the whole cord masks created with JIM as well as the manually segmented GM masks provided in the training set, where the value of each voxel represented the closest distance from the contour. The distance maps from each slice of the 20 files in the training set were separated by level and then used to create templates for the overall cross-sectional shape of the whole cord and templates of the cross-sectional spinal cord grey-matter shape. The registration software used was an internally developed tool (Carballido-Gamio et al., 2013), which was programmed in MATLAB (The Mathworks, Inc. Natick, MA) to enable distance map based registrations (Reinertsen et al., 2004, Suh and Wyatt, 2006).

### Creating an initial guess for grey matter segmentation based on registration

To segment the GM in an image of the spinal cord, an initial guess of the segmentation must be provided to the active contours algorithm. This initial guess is based on the non-linear transformation of the previously created level specific whole cord template to the delineated whole cord in the image slice. The computed affine and non-linear transformations are then applied to the previously created spinal cord GM template. The transformed GM template gives a rough idea of the GM segmentation in each subject.

### Morphological geodesic active contour model

The registered GM template is then used as an initial guess to initialize the geodesic active contour algorithm. Traditional active contour models are methods used in computer vision where a deformable spline is warped, subject to certain constraints and image forces, until a predefined overall energy is minimized (Kass et al., 1988). The standard solution for contour evolution algorithms involves numerical methods of integration that are computationally costly and may have issues with stability. The original active contours approach also depends on the parametrization of the contour and has trouble handling changes in curve topology. The geodesic active contour method addresses these issues, reduces the need for preprocessing since it utilizes fewer parameters, and is better able to recognize an object with non-ideal edges (Caselles et al., 1997). Recently, a new approach (Márquez-Neila et al., 2014) that utilizes morphological operators with a geodesic active contour method was developed that allows for a much faster and more stable process of contour evolution. The MGAC algorithm makes use of a publicly available Python implementation of the morphological geodesic active contour method (http://github.com/pmneila/morphsnakes). To use this implementation, the user provides an initial contour which is then deformed in a method driven by three image forces: a smoothing force that controls the smoothness of the contour, a balloon force that inflates or deflates the contour in areas where information is lacking, and an image attraction force, which drives the contour to the maximum gradient areas in the image. Our parameters were selected according to the methods and guidelines stated in the study that developed this morphological geodesic active contour method (Márquez-Neila et al., 2014). A comparison of MGAC results with manual segmentations is shown in Fig. 7.

## Grey matter segmentation based on maximum entropy

The Grey matter Segmentation Based on Maximum Entropy (GSBME) algorithm is a three-stage procedure for the semi-automatic, supervised segmentation of the GM in anatomical magnetic resonance images of the human spinal cord. Fig. 8 summarises the three stages of the GMSE algorithm. The first stage is pre-processing; the second stage is the thresholding of the sum of grey/white matter signal intensity entropies; the final stage represents an outlier detector. Details of each stage are provided below. All stages were implemented in MATLAB${}^{®}$ 2015a (The MathWorks, Inc., Natick, Massachusetts, USA), unless otherwise stated.

### Preprocessing

The aim of the pre-processing stage is to detect the spinal cord and to increase the quality of the data for the thresholding stage. Additionally, a step of signal normalisation was also implemented, as for the challenge images from different sites were provided in different ranges. Specifically, in the pre-processing stage, the following steps were carried out.

1. The spinal cord was detected with the Spinal Cord Toolbox *sct*_*propseg* command (de Leener et al., 2014), using manual initialisation.
2. Signal intensities were normalised slice-by-slice as $u=\frac{u_{L}+(u_{H}-u_{L})}{\left(s_{H}-s_{L}\right)}(s-s_{L})$, setting *u*_*L*_=0.2, *u*_*H*_=0.7. Above, *s* is the intensity in the non-normalised image, while *s*_*L*_ and *s*_*M*_ ($s_{L}<s_{M}$) are the two means obtained fitting a two-component Gaussian mixture model (GMM). The GMM was fitted to the intensity values of the spinal cord in each slice after filtering the image with a 2D median filter (3×3 voxel×voxel).
3. The normalised images were denoised slice-by-slice with Split Bergman isotropic total variation approach (Goldstein and Osher, 2009). A freely available MATLAB implementation was used,^1^ setting the weight of the regularising term to μ=0.15.

### Thresholding

The denoised images were thresholded slice-by-slice with the aim of identifying signal hyperintensities likely to be GM. The thresholding was performed within a sliding window whose size was defined as $1.5\sqrt{\pi^{-1}A}$, where A is the cord area, evaluated from the cord mask. The optimal threshold $T^{*}$ for each position of the sliding window was obtained maximising the sum of grey and white matter signal intensity entropies, i.e. $T^{*}=\arg\;\underset{T}{\max}\;\;\;H(T)$, with *H*(*T*) calculated as

(10) $$H(T)=\overset{\;}{\underset{u}{\sum }}-\;p(u\;|\;u<T)\log_{2}\left(p(u\;|\;u<T)\right)\;\;+\;\;\;\ldots \ldots \;\;\;+\;\;\;\overset{\;}{\underset{u}{\sum }}-\;p(u\;|\;u\geq T)\log_{2}\left(p(u\;|\;u\geq T)\right).$$

Above, the first and second summations represent respectively the entropy of the white and GM signal, while *u* is the signal intensity after normalisation and denoising. Prior to thresholding, the map of maximum-entropy thresholds was smoothed with a Gaussian filter.

### Outlier detection

The last stage consisted of an outlier detector that discards segmented hyperintensities depending on their morphological features, implemented with the Data Description Toolbox *DDTools*^2^ (Tax, 2015). The features were morphological characteristics derived from each candidate hyperintense region: major/minor axis length; equivalent diameter; perimeter; eccentricity; filled area; extent; solidity; weighted/unweighted centroid; seven invariant moments (Hu, 1962). Features were normalised in [−1;+1], and a one-class Gaussian model was trained on the binary segmentations of the GM from the training data (majority voting of the raters), with a target error of 2%. Data were resampled at the same resolution for this particular step.

## Spinal cord toolbox

The proposed GM segmentation as implemented in the Spinal Cord Toolbox (SCT) (de Leener, 2016), is based on multi-atlas segmentation and was built from previous work (Asman and Bryan, 2014), and includes additional features to improve robustness (vertebral level information) and applicability to other contrasts (via intensity normalization) (Dupont, 2016).

### Model construction

The model is constructed out of a dataset of WM/GM contrasted images and manual segmentation of the GM (Fig. 9-1). Each image is preprocessed with the following steps: (i) the SC is automatically segmented using PropSeg (de Leener et al., 2014), (ii) the image is resampled to an axial resolution of 0.3×0.3 mm^2^, (iii) and smoothed using a non-local means adaptative algorithm (Manjón et al., 2010), (iv) the image is masked using the SC segmentation, (v) and cropped using a square mask of 75×75 pixels centered on the spinal cord (constituting a rigid pre-registration at the same time), finally (vi) the image is splitted along the rostro-caudal direction and considered slice by slice. After preprocessing, each slice is co-registered to a common space as follows: the WM segmentation (obtained by subtracting the manual GM segmentation to the automatic SC segmentation) is registered to the average WM segmentation (averaged using majority voting label fusion) using an affine transformation (gradient step=0.5, metric=mutual information) as implemented in ANTs (Avants and Tustison, 2014). The same transformation is then applied to the associated image. The intensity of the WM and GM of the image is normalized to the median WM and GM from the dictionary.

The images are then used to perform a principal component analysis (PCA): the dictionary images constitute the original space, in which each dimension corresponds to the variation of intensity in one given pixel among the dictionary slices. To perform the PCA, the covariance matrix of all the dictionary slices is computed, and eigenvectors and eigenvalues are deduced by diagonalization. The eigenvectors are sorted by decreasing eigenvalues and the first eigenvectors explaining 80% of the variability are kept (this value was chosen based on preliminary results). The kept eigenvectors are the dimensions of a reduced space that constitutes the model.

### Image segmentation

The image to segment is first preprocessed following the same 6 steps described above (Fig. 9, Fig. 2). Then, each slice is registered on the dictionary mean image using an affine registration (same parameters as described above). All transformations are stored to be able to apply the inverse transformations to the results of segmentation. To have the method work with any contrast, the intensity of the GM and WM in the image to segment is estimated using the dictionary manual segmentations averaged per vertebral level, then the slice intensity is normalized as described above. Each slice of the image to segment is then projected into the model space (Fig. 9, Fig. 3) and the similarity ($\beta_{i,j}$) between the image slice (*i*) and each dictionary slice (*j*) is computed (Fig. 9, Fig. 4) using the coordinates of the slices in the model space ($w_{i}^{\tau }$ and *w*^*dic*^_*i*_) as well as the vertebral level information of each slice ($l_{i}^{\tau }$ and *l*^*dic*^_*i*_) as described in Eq. (11). With *Z*, the partition function such as $\sum_{j=1}^{J}\beta_{i,j}=1$; *J*, the total number of slices in the dictionary; *γ*, a weighting parameter associated with the vertebral level and empirically set to 2.5; *τ*, a weighting parameter related with the geodesic distance defined by Asman et al. in Eq. (16) of their paper (Asman and Bryan, 2014).

(11) $$\beta_{i,j}=\frac{1}{Z}\exp(-\gamma |l_{i}^{\mathrm{im}}-l_{j}^{\mathrm{dic}}|)\exp(-\tau ∥w_{i}^{\mathrm{im}}-w_{j}^{\mathrm{dic}}{∥}_{2})$$

The dictionary slices the most similar to the image slice are selected (Fig. 9, Fig. 5) using an arbitrary threshold ($E=\frac{1}{2J}$, E cannot be larger than $\frac{1}{J}$ for the sake of the computation of *τ*). Then, the manual GM and WM segmentations associated with selected dictionary slices are averaged using the majority voting label fusion (Fig. 9, Fig. 6). Finally, the automatic segmentations are resampled and registered back into the image original space using the inverse of the preprocessing transformations.

## Semisupervised VBEM

The proposed method (*Semisupervised VBEM*) relies on a quite general Bayesian generative model of structural MRI data, which can potentially be applied to any large MR database.

The purpose of the framework is to capture both shape and intensity variability of MR data, within a population. This is, in fact, a primary research topic, as it permits addressing many problems related to the processing and interpretation medical of imaging data, such as image segmentation (Ashburner and Friston, 2005), structural labeling (Tzourio-Mazoyer et al., 2002) and spatial normalization (Ashburner and Friston, 1999). These processing tasks, in turn, constitute the basis for performing morphometric group studies, which have been extensively used, in the neuroimaging community, for the in-vivo investigation of brain structures, in both physiological and pathological conditions (Ashburner and Friston, 2000, Good et al., 2002).

Within the proposed method, MR signals are treated as observed data generated by warping of an average shaped reference anatomy. The intensity values of the different tissue types are assumed to be drawn from Gaussian Mixture distributions (GM) (Ashburner and Friston, 2005).

In formulas, the probability of observing intensity x at voxel *j*, with *j* belonging to tissue class *k*, can be expressed as

(12) $$p(x_{j},z_{\mathrm{jk}}=1)=\pi_{k}N(x_{j}|\mu_{k},\Sigma_{k})=\overset{K}{\underset{c=1}{\prod }}{\left[\pi_{c}N(x_{j}|\mu_{c},\Sigma_{c})\right]}^{z_{\mathrm{jc}}}\;,$$

where z is a binary latent variable encoding class memberships, $\mu_{k}$ and $\Sigma_{k}$ are the mean and covariance matrix of the *k*th mixture component and *π*_*k*_ represents the prior probability of finding tissue type *k* at voxel *j*. In other words, the prior terms $\left\{\pi_{k}\right\}$ serve to define an average-shaped reference anatomy, in the form of tissue probability maps (TPMs). Additionally, the robustness of the model is augmented by introducing Gaussian-Wishart priors on the intensity distribution parameters $\left\{\mu_{k},\Sigma_{k}\right\}$.

A graphical representation of the model is reported in Fig. 10, which synthetically highlights the conditional dependencies among all variables.

Model fitting is performed with a variational version of the expectation maximization (EM) algorithm (Bishop, 2006, Corduneanu and Bishop, 2001). For every subject of the data set, this involves alternating between computing sufficient statistics of the observed data and updating the Gaussian means and covariance matrices of all tissue classes, so as to maximize a lower bound on the model evidence. Additionally, deformation fields have to be estimated in order to map between the individual and common reference spaces. This is treated as a complementary optimization problem, which can be solved using numerical optimization techniques (such as the Gauss-Newton method) to maximize the same lower bound on the marginal likelihood (evidence) with respect to a set of deformation parameters.

The tissue probability maps encoded in the priors $\left\{\pi_{k}\right\}$ can either be considered as fixed parameters or unknown quantities to be estimated form the data. As opposed to brain atlases, which are widely available for different healthy and pathological populations (Fonov et al., 2011, Thompson et al., 2001, Tang et al., 2010), reliable and unbiased spine templates have only recently started to be proposed (Fonov et al., 2014). Nevertheless, our method can easily be applied in a fully unsupervised fashion. In such a case, the TPMs are automatically built during model fitting, which also ensures that they are best representative of the population of interest. The framework can be made even more robust by incorporating training data with manual labels, thus leading to a semisupervised learning scheme.
